# Evidence for reduced frontal cortical thickness in long-term heavy cannabis users

**DOI:** 10.1192/j.eurpsy.2026.10204

**Published:** 2026-07-31

**Authors:** A. Aquino-Servin, P. Fuentes-Claramonte, N. Hostalet, I. París-Gómez, P. del Olmo-Encabo, M. Madre, X. Roca, F. Panicali, A. Romaguera, M. Á. García-León, P. J. McKenna, E. Pomarol-Clotet

**Affiliations:** 1 FIDMAG Germanes Hospitalàries Research Foundation; 2 Programa de Doctorat en Biomedicina, Universitat de Barcelona; 3 CIBERSAM ISCIII; 4 Mental Health, IR SANT PAU, Hospital de la Santa Creu i Sant Pau; 5 Fundació Hospitalàries Sant Boi; 6 Fundació Hospitalàries Barcelona Nord, Barcelona; 7 Department of Personality, Assessment, and Psychological Treatments, Universidad de Sevilla, Sevilla, Spain

## Abstract

**Introduction:**

Although cannabis use is often regarded as relatively harmless, structural imaging studies have found variable evidence for volume reductions in the hippocampus and orbitofrontal cortex (OFC) among other regions (Lorenzetti et al. Eur Arch Psychiatry Clin Neurosci; 269 59-71; Soleimani et al. Sci Rep 2023; 13 5847; Mashhoon et al. Drug Alcohol Depend. 2015; 155 275-83; Wittemann et al. Eur. Addict. Res. 2021; 27 115-22). These studies have mostly focused on adolescents and young adult users, and studies in long-term, heavy cannabis users, who would be arguably most likely to show structural alterations, are few.

**Objectives:**

The aim of this study is to examine different morphological metrics at the whole-brain level, including volume, surface area and cortical thickness in long-term, heavy cannabis users.

**Methods:**

We carried out 3T structural imaging in 41 cannabis users with ≥10 years of use and ≥5 years of daily consumption (mean age 31.3 ± 6.97 years), and 40 age, sex, and IQ (measured using the Test de Acentuación de Palabras (Del Ser et al 1999 Brain Cogn; 33 343-56), and non-using controls (<10 lifetime uses). Structural MRI data were processed in FreeSurfer (v7.1.1), to provide measures of surface area, and cortical thickness. Whole-brain, non-parametric permutation testing was conducted with cluster-wise correction (p < .001, two-tailed; 5,000 Monte Carlo iterations), and clusters were considered significant at a corrected p of < .05.

**Results:**

The cannabis users showed two clusters of cortical thickness reduction in the right rostral middle frontal cortex (See Fig1) (max=3.37, cluster=657.94 vertices, p=0.01; max=4.46, cluster=531.57 vertices, p=0.02). No differences were observed in cortical volume or surface area.

**Image 1:**
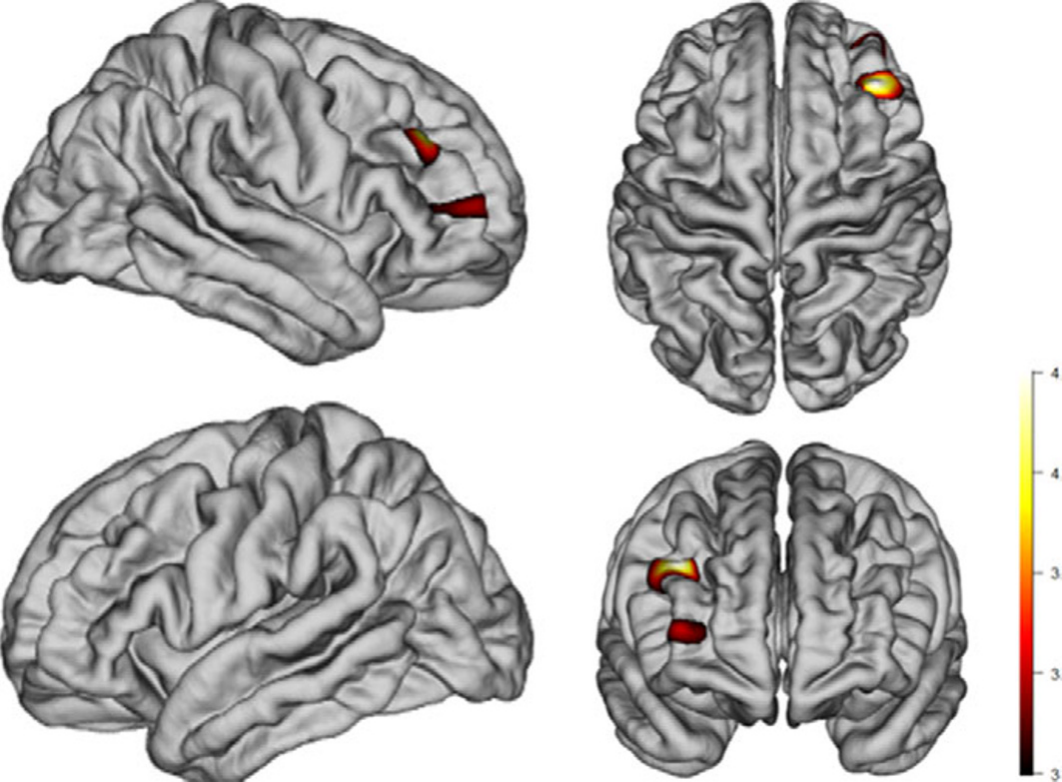
Image 1: Long description.

**Conclusions:**

From this study, chronic cannabis use is associated with cortical thinning in the right rostral middle frontal cortex. Further studies could examine whether these changes relate to the executive and other neuropsychological impairments reported in users.

**Disclosure of Interest:**

None Declared

## Long descriptions

### Image 1: Long description

A four-panel anatomical visualization of the human brain with heat-map activation overlays.

* Top-left panel: Lateral view of the right hemisphere showing two small red activation clusters in the frontal and parietal regions.

* Top-right panel: Superior view (top-down) showing a bright yellow and red activation cluster in the right frontal lobe.

* Bottom-left panel: Lateral view of the left hemisphere with no visible activation clusters.

* Bottom-right panel: Posterior view (back-to-front) showing two activation clusters on the left side of the image, corresponding to the right hemisphere. The upper cluster is bright yellow and red, while the lower is dark red.

* Color scale: A vertical bar on the far right indicates intensity. The scale ranges from dark red at the bottom (value 3) to bright yellow at the top (value 4).

Navigate back to Image 1:.

